# External Validation of a Prediction Model for Acute Kidney Injury Following Noncardiac Surgery

**DOI:** 10.1001/jamanetworkopen.2021.27362

**Published:** 2021-10-18

**Authors:** Masatoshi Nishimoto, Miho Murashima, Maiko Kokubu, Masaru Matsui, Masahiro Eriguchi, Ken-ichi Samejima, Yasuhiro Akai, Kazuhiko Tsuruya

**Affiliations:** 1 Department of Nephrology, Nara Medical University, Kashihara, Nara, Japan; 2 Department of Nephrology, Nagoya City University Graduate School of Medical Sciences, Nagoya, Aichi, Japan; 3 Department of Nephrology, Nara Prefecture General Medical Center, Nara, Nara, Japan

## Abstract

**Importance:**

The Simple Postoperative AKI Risk (SPARK) index is a prediction model for postoperative acute kidney injury (PO-AKI) in patients undergoing noncardiac surgery. External validation has not been performed.

**Objective:**

To externally validate the SPARK index.

**Design, Setting, and Participants:**

This single-center retrospective cohort study included adults who underwent noncardiac surgery under general anesthesia from 2007 to 2011. Those with obstetric or urological surgery, estimated glomerular filtration rate (eGFR) of less than 15 mL/min/1.73 m^2^, preoperative dialysis, or an expected surgical duration of less than 1 hour were excluded. The study was conducted at Nara Medical University Hospital. Data analysis was conducted from January to July 2021.

**Exposures:**

Risk factors for AKI included in SPARK index.

**Main Outcomes And Measures:**

PO-AKI, defined as an increase in serum creatinine of at least 0.3 mg/dL within 48 hours or 150% compared with preoperative baseline value or urine output of less than 0.5 mL/kg/h for at least 6 hours within 1 week after surgery, and critical AKI, defined as either AKI stage 2 or greater and/or any AKI connected to postoperative death or requiring kidney replacement therapy before discharge. The discrimination and calibration of the SPARK index were examined with area under the receiver operating characteristic curves (AUC) and calibration plots, respectively.

**Results:**

Among 5135 participants (2410 [46.9%] men), 303 (5.9%) developed PO-AKI, and 137 (2.7%) developed critical AKI. Compared with the SPARK cohort, participants in our cohort were older (median [IQR] age, 56 [44-66] years vs 63 [50-73] years), had lower baseline eGFR (median [IQR], 82.1 [71.4-95.1] mL/min/1.73 m^2^ vs 78.2 [65.6-92.2] mL/min/1.73 m^2^), and had a higher prevalence of comorbidities (eg, diabetes: 3956 of 51 041 [7.8%] vs 802 [15.6%]). The incidence of PO-AKI and critical AKI increased as the scores on the SPARK index increased. For example, 10 of 593 participants (1.7%) in SPARK class A, indicating lowest risk, experienced PO-AKI, while 53 of 332 (16.0%) in SPARK class D, indicating highest risk, experienced PO-AKI. However, AUCs for PO-AKI and critical AKI were 0.67 (95% CI, 0.63-0.70) and 0.62 (95% CI, 0.57-0.67), respectively, and the calibration was poor (PO-AKI: y = 0.24x + 3.28; *R*^2^ = 0.86; critical AKI: y = 0.20x + 2.08; *R*^2^ = 0.51). Older age, diabetes, expected surgical duration, emergency surgery, renin-angiotensin-aldosterone system blockade use, and hyponatremia were not associated with PO-AKI in our cohort, resulting in overestimation of the predicted probability of AKI in our cohort.

**Conclusions and Relevance:**

In this study, the incidence of PO-AKI increased as the scores on the SPARK index increased. However, the predicted probability might not be accurate in cohorts with older patients with more comorbidities.

## Key Points

**Question:**

Is the Simple Postoperative AKI Risk (SPARK) index, which was developed to predict postoperative acute kidney injury in noncardiac surgery, useful in a different population?

**Findings:**

In a cohort study of 5135 adults in Japan, the incidence of postoperative acute kidney injury increased as scores on the SPARK index increased. However, the model’s discriminative and calibration powers were suboptimal owing to overestimated probability among those with especially high risk of developing acute kidney injury.

**Meaning:**

These findings suggest that it is difficult to precisely predict the probability of acute kidney injury preoperatively in noncardiac surgery, which includes various surgical procedures and participants with various medical backgrounds.

## Teaser

This cohort study externally validates the Simple Postoperative Acute Kidney Injury Risk (SPARK) index for prediction of acute kidney injury among patients undergoing noncardiac surgery.

## Introduction

Postoperative acute kidney injury (PO-AKI) is a serious complication of surgical procedures that is associated not only with short-term increase in the risk of mortality^1,2,3^ but also with long-term mortality.^4,5,6^ Currently, there are no effective treatments for AKI; therefore, it is important to identify patients with high risk preoperatively and manage them with caution to prevent AKI. Preoperative risk stratification is useful for planning risk-based monitoring and management strategies to prevent PO-AKI and AKI-related death or dialysis.

In cardiac surgery, several prediction models have been developed and validated internally or externally.^7^ However, PO-AKI in noncardiac surgery has been less well studied.

Recently, a novel model, the Simple Postoperative AKI Risk (SPARK) index,^8^ was developed in noncardiac surgery in Korea. The performance of the SPARK index was externally validated in another cohort, and the discriminative and calibration powers of the model were reasonable. However, external validation was performed in a cohort from a different hospital that is affiliated with the same university hospital, where patient population and clinical practice patterns were likely to be similar. Thus, further external validation in a different population is warranted. The aim of the present study was to validate the SPARK index for PO-AKI in the NARA-AKI cohort, a retrospective cohort of AKI following noncardiac surgery in Japan.^9,10,11,12^

## Methods

### Source of Data and Participants

The NARA-AKI cohort study is a single center, retrospective cohort study. Inclusion criteria were as follows: patients aged at least 18 years who underwent noncardiac surgery under general anesthesia from April 2007, when electronic medical records started at our hospital, to December 2011 at Nara Medical University Hospital. Exclusion criteria were those who underwent cardiac surgery, obstetric surgery (as serum creatinine levels decrease during pregnancy due to hemodilution and criteria for AKI are not validated in pregnant women), urological surgery (as increase in creatinine due to nephrectomy or ureteral manipulation could have different underlying mechanisms than PO-AKI), preoperative dialysis; those with missing data for serum creatinine within 1 month before and 1 week after surgery; and those with other missing values for analyses. In addition, those with an estimated glomerular filtration rate (eGFR) of less than 15 mL/min/1.73 m^2^ or an expected surgical duration of less than 1 hour were excluded, as in the SPARK study.^8^ Only the first eligible surgery was considered if participants underwent multiple surgeries during the study period. The study protocol and waiver of consent were approved by Nara Medical University Ethics Committee. This study waived the requirement for written informed consent due to its retrospective nature. Rather, research content was included on our department’s web page.^13^ This study was conducted in accordance with the Declaration of Helsinki.^14^ The study was registered in the University hospital Medical Information Network (UMIN000037141). This study adhered to the Transparent Reporting of a Multivariable Prediction Model for Individual Prognosis or Diagnosis (TRIPOD) reporting guideline.

### Predictors

Predictors were the variables included in SPARK index.^8^ They were age, sex, diabetes, renin-angiotensin-aldosterone-system (RAAS) blockade (angiotensin-converting enzyme inhibitors and/or angiotensin receptor blockers), preoperative eGFR, dipstick albuminuria, anemia, hyponatremia, hypoalbuminemia, expected surgical duration (hours), and emergent surgery.

### Outcomes

The outcome variables were PO-AKI and critical AKI. PO-AKI was defined according to Kidney Disease Improving Global Outcomes criteria^15^ (increase in serum creatinine ≥0.3 mg/dL within 48 hours [to convert to micromoles per liter, multiply by 88.4] or 150% compared with preoperative baseline value or urine output <0.5 mL/kg/h for ≥6 hours within 1 week after surgery). Critical AKI was defined as either AKI stage 2 or greater and/or any AKI connected to postoperative death or requiring kidney replacement therapy before discharge.

### Data Acquisition and Definition

The list of participants who underwent noncardiac surgery under general anesthesia, age, sex, date of surgery, and laboratory data were automatically abstracted from electronic medical records. Comorbidities, use of medications, expected surgery duration, and outcomes were manually searched from electronic medical records by investigators. These data were obtained from detailed medical record reviews, including inquiry on prescriptions from other medical facilities. Chart review was performed by M.N., M. Murashima, and M.K., using the same data abstraction sheet and definitions of each variable. A random selection of 100 samples was cross-checked by abstractors. We confirmed that there were no discrepancies between abstractors. Baseline laboratory data, including serum creatinine level, were defined as values within 1 month before surgery and the closest to the date of surgery. eGFR was calculated using the equation developed for Japanese populations by the Japanese Society of Nephrology, based on the baseline serum creatinine value.^16^ Age was stratified into younger than 40 years, from 40 and to younger than 60 years, from 60 and younger than 80 years, and 80 years and older. Preoperative eGFR was classified into at least 60 mL/min/1.73 m^2^, from 45 and less than 60 mL/min/1.73 m^2^, from 30 and less than 45 mL/min/1.73 m^2^, and from 15 and less than 30 mL/min/1.73 m^2^. Expected surgical duration was abstracted from an application form submitted by surgeons preoperatively for scheduling the operation. Hypoalbuminemia was defined as serum albumin levels less than 3.5 g/dL (to convert to grams per liter, multiply by 10); anemia, hemoglobin levels less 13 g/dL for men or less than 12 g/dL for women (to convert to grams per liter, multiply by 10). Hyponatremia was defined as a serum sodium concentration of less than 135 mEq/L (to convert to millimoles per liter, multiply by 1.0). Albuminuria was defined as dipstick albuminuria of at least 1. All these cutoff values were derived from the SPARK index study.^8^ In this study, types of surgery were originally divided into 4 categories (ie, intrathoracic surgery, intra-abdominal surgery, pelvic or major joint surgery, and other types of surgery) in accordance with a previous study^17^ and recategorized for the validation of the SPARK index.

### Statistical Analysis

The data were expressed as mean with SD, median with IQR, or number with a percentage, as appropriate. First, risk scores for AKI were calculated according to the SPARK index for each participant in our cohort, and the participants were classified into 4 categories of SPARK class (A, indicating lowest risk, to D, indicating highest risk). The incidence rates of PO-AKI and critical AKI in each class were assessed. Second, the discrimination ability of the SPARK index was evaluated with area under the receiver operating characteristic (ROC) curve (AUC). An AUC of at least 0.7 was regarded as acceptable. Third, calibration was examined by plotting predicted probability against observed probability. In the SPARK study, model coefficients were multiplied by 11.0306 and rounded to integers to construct the SPARK index. To calculate predicted probability in our cohort, regression coefficients for each variable in the SPARK index were recalculated from scores in the index divided by 11.0306. The intercepts for ordinal logistic regression were 5.9651 for PO-AKI and 7.7281 for critical AKI (provided by the main authors of the original manuscript). Discriminative and calibration powers were reassessed after the imputation of missing covariates by multiple imputation by chained equation method using all the variables included in the SPARK index. For imputation of continuous variables, binary outcomes, and categorical variables, predictive mean matching, logistic regression, and multinomial logistic regression were used, respectively. Five imputed data sets were created. Fourth, ordinary logistic regression was performed using variables included in the final SPARK index to examine whether these variables were also significantly associated with AKI in our cohort. Additionally, model updating^18^ was performed and assessed by cross-validation. The NARA-AKI cohort was randomly divided into 2 groups (2:1 ratio) to create the new derivation and validation cohorts. Each coefficient of all the covariates included in SPARK index and intercept were calculated in the new derivation cohort. Predicted probability of PO-AKI and critical AKI was calculated from regression models in the derivation cohort, and the discriminative and calibration powers were reassessed in the validation cohort. In addition, internal validation was performed by bootstrap methods using logistic regression. Values of *P* < .05 were considered statistically significant, and all tests were 2-tailed. Statistical analyses were performed using Stata version 15 (StataCorp).

## Results

### Participants

**Figure 1.**
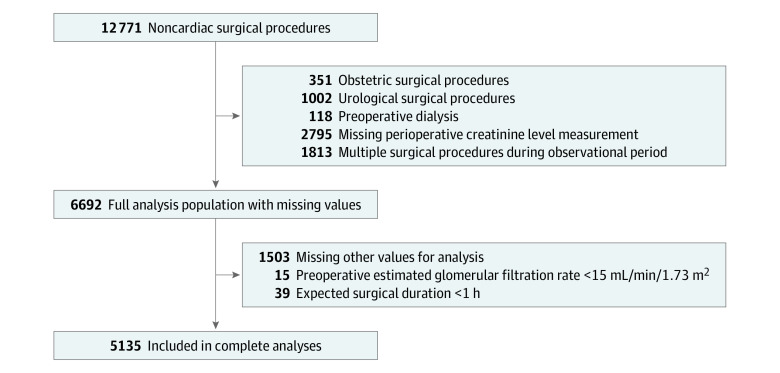
Study Flowchart

During the study period, 12 771 patients underwent noncardiac surgeries under general anesthesia at Nara Medical University Hospital. After the exclusion of data by exclusion criteria, data for 5135 participants were available for analyses (Figure 1). In the NARA-AKI cohort, the median (IQR) age was 63 (50-73) years, and men accounted for 46.9% of the cohort (2410 participants). The median (IQR) eGFR was 78.2 (65.6-92.2) mL/min/1.73 m^2^ (Table 1). Compared with participants in the discovery cohort of SPARK study, those in our cohort were older (median [IQR] age, 56 [44-66] years vs 63 [50-73] years), had a higher prevalence of diabetes (3956 of 51 041 [7.8%] vs 802 [15.6%]) and hypertension (9824 [19.2%] vs 1817 [35.4%]), had a lower eGFR (median [IQR], 82.1 [71.4-95.1] mL/min/1.73 m^2^ vs 78.2 [65.6-92.2] mL/min/1.73 m^2^), and had a longer expected surgical duration (median [IQR], 2.5 [2.0-3.0] hours vs 3.0 [2.5-5.0]). The proportion of participants regularly taking RAAS blockade were higher in this cohort than in the SPARK discovery cohort (963 [18.8%] vs 2881 [5.6%]) (eTable 1 in the Supplement). Among 5135 participants in this study, 303 (5.9%) and 137 (2.7%) developed PO-AKI and critical AKI, respectively.

**Table 1.** Participant Characteristics

Characteristic	Participants, No. (%) (N = 5135)
Age, y	
Median (IQR)	63 (50-73)
<40	746 (14.5)
≥40 to <60	1326 (25.8)
≥60 to <80	2681 (52.2)
≥80	382 (7.4)
Men	2410 (46.9)
Women	2725 (53.1)
Body mass index, median (IQR)^a^	22.5 (20.3-24.9)
Preexisting comorbidities	
Heart disease^b^	298 (5.8)
Hypertension	1817 (35.4)
Diabetes	802 (15.6)
Types of surgery^c^	
General	4323 (84.2)
Urologic	0
Obstetrics	0
Pelvic or major joint surgery	812 (15.8)
Surgery duration, median (IQR), h	2.8 (1.9-4.1)
Expected surgery duration, median (IQR), h	3.0 (2.5-5.0)
Anesthesia type	
General	5135 (100)
Nongeneral	0
Emergency surgery	292 (5.7)
Blood pressure before surgery, median (IQR), mm Hg^d^	
Systolic	140 (125-160)
Diastolic	80 (70-85)
RAAS blockade use	963 (18.8)
Laboratory findings	
eGFR, median (IQR), mL/min/1.73 m^2^	78.2 (65.6-92.2)
CKD stage	
None, 1, or 2 (eGFR ≥60 mL/min/1.73 m^2^)	4278 (83.3)
3A (eGFR ≥45 to <60 mL/min/1.73 m^2^)	590 (11.5)
3B (eGFR ≥30 to <45 mL/min/1.73 m^2^)	184 (3.6)
4 (eGFR ≥15 to <30 mL/min/1.73 m^2^)	83 (1.6)
Dipstick albuminuria	470 (9.2)
White blood cell count, median (IQR), /μL	6000 (4900-7500)
Hemoglobin, mean (SD), g/dL	12.8 (1.9)
Anemia	1902 (37.0)
Platelet count, median (IQR), 10^3^/μL	231 (190-280)
Albumin, mean (SD), g/dL	4.2 (0.5)
Hypoalbuminemia	470 (9.2)
Sodium, mean (SD), mEq/L	141 (2.9)
Hyponatremia	149 (2.9)
Potassium, mean (SD), mEq/L	4.1 (0.4)

Abbreviations: CKD, chronic kidney disease; eGFR, estimated glomerular filtration rate; RAAS, renin-angiotensin-aldosterone system.

SI conversion factors: To convert white blood cell count to ×10^9^ per liter, multiply by 0.001; hemoglobin to grams per liter, multiply by 10.0; platelets to ×10^9^ per liter, multiply 1.0; albumin to grams per liter, multiply 10.0; sodium and potassium to millimoles per liter, multiply 1.0.

^a^ Body mass index was calculated as weight in kilograms divided by height in meters squared.

^b^ Heart disease was defined as the history of heart failure or coronary artery disease (angina or myocardial infarction).

^c^ In the NARA-AKI cohort, types of surgery were originally divided into 4 categories (ie, intra-thoracic surgery, intra-abdominal surgery, pelvic or major joint surgery, and other types of surgery), and recategorized for the validation of SPARK index. Gynecological surgery, such as total hysterectomy, and orthopedic surgery, including knee or hip replacement, were classified as pelvic or major joint surgery. Other surgical procedures, including intra-thoracic and intra-abdominal surgical procedures as well as neurosurgery, were classified as general surgery.

^d^ Systolic and diastolic blood pressure were measured in the operation room before induction of anesthesia.

### Model Performance

**Figure 2.**
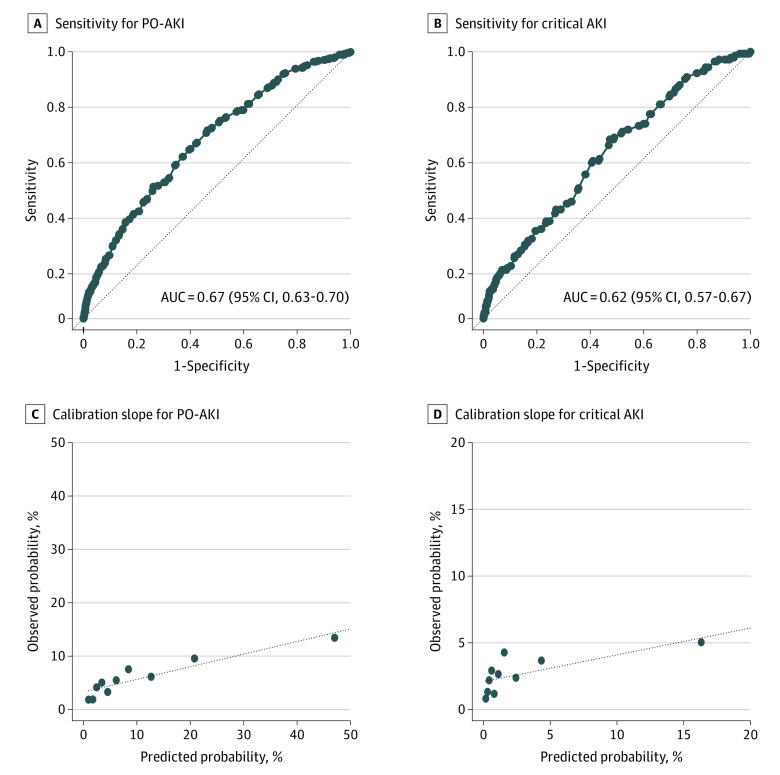
Receiver Operating Characteristic Curves and Calibration Plots for Postoperative Acute Kidney Injury (PO-AKI) and Critical AKI AUC indicates area under the curve.

In our cohort, a higher proportion of participants were classified into SPARK class C and D and the incidences of AKI in our cohort were higher in higher classes of SPARK classification compared with participants in the SPARK discovery cohort (Table 2; eTable 2 in the Supplement). Overall, 10 of 593 participants (1.7%) in SPARK class A experienced PO-AKI, while 53 of 332 (16.0%) in SPARK class D experienced PO-AKI. However, the incidence of AKI was lower in our cohort among those classified as SPARK class C and D compared with the SPARK cohort (Table 2; eTable 2 in the Supplement). The ROC curves for PO-AKI and critical AKI appear in Figure 2A and 2B. AUCs for PO-AKI and critical AKI were 0.67 (95% CI, 0.63-0.70) and 0.62 (95% CI, 0.57-0.67), respectively. The calibration slopes were y = 0.24x + 3.28 (*R*^2^ = 0.86) for PO-AKI and y = 0.20x + 2.08 (*R*^2^ = 0.51) for critical AKI, which suggested poor calibration (Figure 2C and 2D). Multiple imputation was performed in a data set of 6692 participants, including those with missing data. Sensitivity analyses using imputed data sets yielded similar results. After excluding those with an eGFR of less than 15 mL/min/1.73 m^2^ or with expected surgical duration of less than 1 hour, AUCs for PO-AKI and critical AKI were 0.66 and 0.64, respectively. The calibration slopes were y = 0.24x + 3.62 (*R*^2^ = 0.89) for PO-AKI and y = 0.29x + 2.11 (*R*^2^ = 0.81) for critical AKI.

**Table 2.** Incidence of AKI Stratified by SPARK Classes

SPARK class	Total score	Participants, No. (%)	
		Incidence of PO-AKI	Incidence of critical AKI
A	<20	10/593 (1.7)	4/593 (0.7)
B	≥20 to <40	119/2711 (4.4)	67/2711 (2.5)
C	≥40 to <60	121/1499 (8.1)	43/1499 (2.9)
D	≥60	53/332 (16.0)	23/332 (6.9)

Abbreviations: AKI, acute kidney injury; SPARK, Simple Postoperative AKI Risk; PO-AKI, postoperative acute kidney injury.

### Model Updating

Comparison of the model coefficients between the discovery cohorts of the SPARK index and ours is shown in Table 3 and eTable 3 in the Supplement. In our cohort, sex, eGFR, anemia, hypoalbuminemia, and albuminuria were significantly associated with AKI. Regression coefficients for these variables were similar to those in SPARK index except for male sex; it was smaller in our cohort. On the other hand, age, diabetes, expected surgical duration, emergency surgery, RAAS blockade use, and hyponatremia were not associated with PO-AKI and critical AKI in our cohort. As the proportion of participants with older age, diabetes, longer expected surgical duration, emergency surgery, and RAAS blockade use were higher in our cohort, these differences in the associations between variables in the SPARK index and AKI resulted in an overestimation of the probability of AKI in our cohort. The cross-validation of the model constructed in NARA-AKI cohort resulted in better calibration for PO-AKI (y = 0.92x + 0.08; *R*^2^ = 0.88) and critical AKI (y = 1.23x − 0.77; *R*^2^ = 0.98); however, discriminative powers remained suboptimal for PO-AKI (AUC, 0.69; 95% CI, 0.64-0.75) and critical AKI (AUC, 0.65; 95% CI, 0.57-0.73) (eFigure, A and B, in the Supplement). In addition to cross-validation, internal validation by bootstrap method was performed using logistic regression models. Using 200 resamples, the optimism-corrected AUCs for PO-AKI and critical AKI were 0.69 (95% CI, 0.66-0.72) and 0.64 (95% CI, 0.60-0.69), respectively.

**Table 3.** Regression Coefficients in the Proportional Odds Model

Factor	Coefficient (95% CI)
Age, y (vs <40)	
≥40 to <60	0.272 (−0.195 to 0.740)
≥60 to <80	0.178 (−0.269 to 0.625)
≥80	0.064 (−0.513 to 0.642)
Men (vs women)	0.341 (0.098 to 0.584)^a^
Diabetes (vs none)	0.238 (−0.058 to 0.535)
Expected surgical duration (continuous, h)	0.032 (−0.032 to 0.096)
Emergency surgery (vs planned)	0.012 (−0.450 to 0.473)
RAAS blockade use (vs none)	0.123 (−0.170 to 0.422)
eGFR (vs ≥60 mL/min/1.73 m^2^)	
≥45 to <60 mL/min/1.73 m^2^	0.446 (0.099 to 0.794)^a^
≥30 to <45 mL/min/1.73 m^2^	1.143 (0.706 to 1.579)^a^
≥15 to <30 mL/min/1.73 m^2^	1.267 (0.699 60 1.835)^a^
Anemia (vs none)	0.399 (0.131 to 0.666)^a^
Hypoalbuminemia (vs none)	0.740 (0.399 to 1.081)^a^
Hyponatremia (vs none)	0.071 (−0.486 to 0.627)
Albuminuria (vs none)	0.505 (0.173 to 0.836)^a^

Abbreviations: eGFR, estimated glomerular filtration rate; RAAS, renin-angiotensin-aldosterone system.

^a^ *P* < .05.

## Discussion

Our validation study demonstrated that the incidence of PO-AKI increased as the scores on the SPARK index increased. On the other hand, the discriminative and calibration powers of SPARK index for PO-AKI and critical AKI were suboptimal. Model updating in the NARA-AKI cohort resulted in poor improvement in discrimination.

The main cause of poor discrimination and calibration in our cohort was the different associations of the risk factors included in SPARK index with AKI in their cohort vs our cohort. Among the variables included in the SPARK index, age, diabetes, expected surgical duration, emergency surgery, RAAS blockade use, and hyponatremia were not associated with PO-AKI and critical AKI in our cohort. In our cohort, participants were older, had a higher prevalence of diabetes, and had a longer expected surgical duration. The proportions of participants with regular preoperative use of RAAS blockades or those who underwent emergency surgery were also higher. As the result, the proportions of participants classified as SPARK class C or D was higher in our cohort than in that of the SPARK study. Also, predicted probability derived from SPARK index overestimated the observed probability of AKI in our cohort.

There are some possible explanations for the discrepancy in associations between the 2 cohorts. First, some possible risk factors included in the SPARK study might not apply to a different patient population. According to previous studies, whether preoperative use of RAAS blockade is associated with AKI in noncardiac surgery is still being debated^19,20,21,22,23^. RAAS blockades might be associated with AKI only in certain populations or in certain surgical procedures. Furthermore, whether patients continued or stopped taking RAAS blockades on the day of surgery might have affected results. In our cohort, 94 of 963 participants who regularly used RAAS blockade (9.8%) continued the agents on the day of surgery. On the other hand, whether patients did not use the agents on the day of surgery in the SPARK study was not reported. The same would be also true of diabetes^20,24^ and the severity of diabetes; whether insulin therapy had been warranted or not might modify the association with AKI.^25,26^ In addition, older age^17,19,25,27^ and emergency surgery^17,25^ were associated with postoperative AKI in previous studies on noncardiac surgery, whereas neither were associated with AKI in our cohort. These data suggest that there might be effect modifications by patient background for associations between potential risk factors and AKI. Noncardiac surgery includes various surgical procedures, and patients are heterogenous. For example, young patients without comorbidities might undergo cholecystectomy while patients who have heavy smoking habits and atherosclerotic cardiovascular diseases might undergo lung resection for lung cancer. This is in contrast with cardiac surgery, where surgical procedures are mainly limited to coronary artery bypass graft surgery and valvular replacement. Participants undergoing cardiac surgery are usually older and have multiple comorbidities, such as diabetes and hypertension. In cardiac surgery, external validation for prediction models for AKI showed reasonable discrimination power.^7^ Various surgical procedures and heterogenous patient populations in noncardiac surgery might make it difficult to develop a universal prediction model for AKI because of different associations between potential risk factors and AKI. Second, the fact that updated the model resulted in poor improvement in discrimination would suggest the presence of other important predictors. It is difficult to predict AKI with only preoperative information given that intra-operative complications and their management can affect the development of AKI. The SPARK index was intended to perform risk stratification preoperatively and thus used only preoperative variables. However, multiple studies have demonstrated the association of intra-operative factors, such as fluid balance or the use of vasopressors (which are likely reflection of unexpected bleeding or hypotension) with PO-AKI.^9,20,22,24,28,29,30^ In our cohort, participants were older and had more comorbidities than those in SPARK index study. The risk of developing unexpected complications during surgery was likely higher. Third, the methodological differences between the 2 cohorts could additionally contribute to the discrepancy. Urological and obstetric surgeries and surgery under non–general anesthesia, which were included in SPARK study, were excluded from the NARA-AKI cohort, and no data related to these types of surgery were obtained. The increase in serum creatinine levels from nephrectomy and ureteral manipulation could be a different underlying mechanism than other PO-AKI, and the definition of AKI was not validated in an obstetric population. Actually, the subgroup analysis in urological surgery in the SPARK study resulted in poor performance of the index.^8^ This suggests that the exclusion of urological surgery did not result in the poor performance of the SPARK index in our cohort. There are some strengths to our study. To the best of our knowledge, this is the first study to examine the external validity of the novel SPARK index in a large cohort of participants in a different medical community. The observational periods in our study overlapped with the SPARK study, and changes in the care of surgical patients over time would not affect the validity of the model.

### Limitations

This study has limitations. Due to some methodological differences, such as different classification of surgeries, we were not able to perform subgroup analyses.

## Conclusions

In this study, the incidence of PO-AKI increased as scores on the SPARK index increased. However, discriminative and calibration powers were suboptimal. The probability estimated from the SPARK model might not be accurate in cohorts with older participants with more comorbidities. This study highlighted the difficulty of precisely predicting the probability of AKI preoperatively in noncardiac surgery, including various surgical procedures and patients with various medical backgrounds.

## Notes

Supplement. **eTable 1.** Characteristics of Participants **eTable 2.** Comparison of the Incidence of AKI Between NARA-AKI Cohort Study and SPARK Index, Stratified by SPARK Classes **eTable 3.** Comparison of the Regression Coefficients Between NARA-AKI Cohort and the Discovery Cohort of SPARK Index in the Proportional Odds Model **eFigure.** The Receiver Operating Characteristic Curves and Calibration Plots for Postoperative Acute Kidney Injury and Critical Acute Kidney Injury From Cross-Validation in NARA-AKI Cohort Study
