# Supplementary material for: External Validation of a Prediction Model for Acute Kidney Injury Following Noncardiac Surgery
Source: JAMA Netw Open. 2021 Oct 18;4(10):e2127362. doi: 10.1001/jamanetworkopen.2021.27362 (PMC8524308; doi:10.1001/jamanetworkopen.2021.27362)
Supplement: Supplement. — eTable 1. Characteristics of Participants eTable 2. Comparison of the Incidence of AKI Between NARA-AKI Cohort Study and SPARK Index, Stratified by SPARK Classes eTable 3. Comparison of the Regression Coefficients Between NARA-AKI Cohort and the Discovery Cohort of SPARK Index in the Proportional Odds Model eFigure. The Receiver Operating Characteristic Curves and Calibration Plots for Postoperative Acute Kidney Injury and Critical Acute Kidney Injury From Cross-Validation in NARA-AKI Cohort Study [file jamanetwopen-e2127362-s001.pdf]

Supplemental Online Content

Nishimoto M, Murashima M, Kokubu M, et al. External validation of a prediction model for acute kidney injury following noncardiac surgery. *JAMA Netw Open.* 2021;4(10):e2127362. doi:10.1001/jamanetworkopen.2021.27362

eTable 1. Characteristics of Participants

eTable 2. Comparison of the Incidence of AKI Between NARA-AKI Cohort Study and SPARK Index, Stratified by SPARK Classes

eTable 3. Comparison of the Regression Coefficients Between NARA-AKI Cohort and the Discovery Cohort of SPARK Index in the Proportional Odds Model

eFigure. The Receiver Operating Characteristic Curves and Calibration Plots for Postoperative Acute Kidney Injury and Critical Acute Kidney Injury From Cross-Validation in NARA-AKI Cohort Study

This supplemental material has been provided by the authors to give readers additional information about their work.

© 2021 Nishimoto M et al. *JAMA Network Open*.

eTable 1. Characteristics of Participants

	No. (%)	
	NARA-AKI cohort (*n*=5135)	SPARK discovery cohort[1] (*n*=51,041)
Age, median (IQR), years	63 (50–73)	56 (44–66)
<40	746 (14.5)	9206 (18.0)
≥40 to <60	1326 (25.8)	20,877 (40.9)
≥60 to <80	2681 (52.2)	19,684 (38.6)
≥80	382 (7.4)	1274 (2.5)
Sex, men	2410 (46.9)	22,735 (44.5)
Body mass index, median (IQR), kg/m^2	22.5 (20.3–24.9)	23.8 (21.7–26.0)
Preexisting comorbidities		
Heart disease[2]	298 (5.8)	1629 (3.2)
Hypertension	1817 (35.4)	9824 (19.2)
Diabetes	802 (15.6)	3956 (7.8)
Types of surgery		
General surgery	4323 (84.2)	22,447 (44.0)
Neurosurgery		5063 (9.9)
Urologic surgery	0 (0)	4265 (8.4)
Obstetrics	0 (0)	7894 (15.5)
Gynecology	812 (15.8)[3]	
Orthopedics		11,372 (22.3)
Surgery duration, median (IQR), hours	2.8 (1.9–4.1)	2.2 (1.5–3.3)
Expected surgery duration, median (IQR), hours	3.0 (2.5–5.0)	2.5 (2.0–3.0)
Anesthesia type		
General	5135 (100)	43,921 (86.6)
Non-general	0 (0)	6789 (13.4)
Emergency surgery	292 (5.7)	732 (1.4)
BP before surgery, median (IQR), mm Hg		
Systolic BP	140 (125–160)[4]	124 (113–135)
Diastolic BP	80 (70–85)[4]	77 (70–85)
Medication		
RAAS blockade use	963 (18.8)	2881 (5.6)

© 2021 Nishimoto M et al. *JAMA Network Open.*

Laboratory findings		
eGFR, median (IQR), mL/min per 1.73 m^2	78.2 (65.6–92.2)	82.1 (71.4–95.1)
No CKD or CKD stage 1 or 2 (≥60)	4278 (83.3)	46,971 (92.0)
CKD stage 3A (≥45 to <60)	590 (11.5)	3226 (6.3)
CKD stage 3B (≥30 to <45)	184 (3.6)	641 (1.3)
CKD stage 4 (≥15 to <30)	83 (1.6)	203 (0.4)
Dipstick albuminuria (≥1+)	470 (9.2)	4682 (9.3)
White blood cell count, median (IQR), /μL	6000 (4900–7500)	6100 (5000–7500)
Hemoglobin, median (IQR), g/dL	12.9 (11.6–14.1)	13.2 (12.1–14.4)
Anemia (hemoglobin <12 for women, <13 g/dL for men)	1902 (37.0)	14,177 (27.8)
Platelet, median (IQR), 10^3/μL	231 (190–280)	200 (152–256)
Albumin, median (IQR), g/dL	4.3 (4.0–4.5)	4.2 (3.9–4.5)
Hypoalbuminemia (albumin <3.5 g/dL)	470 (9.2)	5148 (10.1)
Sodium, median (IQR), mEq/L	141 (140–143)	140 (139–142)
Hyponatremia (<135 mEq/L)	149 (2.9)	1291 (2.5)
Potassium, median (IQR), mEq/L	4.1 (3.9–4.3)	4.2 (4.0–4.4)

[1]Data from SPARK index study are shown for comparison.

[2]Heart disease was defined as the history of heart failure or coronary artery disease (angina or myocardial infarction).

[3]In NARA-AKI cohort study, types of surgery were originally divided into four categories (i.e. intra-thoracic surgery, intra-abdominal surgery, pelvic or major joint surgery, and other types of surgery) and re-categorized for the validation of SPARK index. Gynecological surgery such as total hysterectomy and orthopedic surgery including knee or hip replacement were classified into pelvic or major joint surgery. Other surgeries including intra-thoracic,

© 2021 Nishimoto M et al. *JAMA Network Open*.

intra-abdominal surgeries were classified into general surgery.

[4]Systolic and diastolic blood pressure were measured in the operation room before induction of anesthesia.

Abbreviations: BP, blood pressure; RAAS, renin-angiotensin-aldosterone system; eGFR, estimated glomerular filtration rate; CKD, chronic kidney disease.

SI conversion factors: To convert hemoglobin to g/L, multiply by 10.0; platelet to 10^9/L, multiply 1.0; albumin to g/L, multiply 10.0; sodium to mmol/L, multiply 1.0; potassium to mmol/L, multiply 1.0.

© 2021 Nishimoto M et al. *JAMA Network Open*.

eTable 2. Comparison of the Incidence of AKI Between NARA-AKI Cohort Study and SPARK Index, Stratified by SPARK Classes

		No. (%)			
SPARK class	Total score	Incidence of AKI		Incidence of critical AKI	
		NARA-AKI cohort	SPARK discovery cohort[1]	NARA-AKI cohort	SPARK discovery cohort[1]
A	<20	10/593 (1.7)	104/12,922 (0.8)	4/593 (0.7)	14/12,922 (0.1)
B	≥20 to <40	119/2711 (4.4)	1182/30,178 (3.9)	67/2711 (2.5)	220/30,178 (0.7)
C	≥40 to <60	121/1499 (8.1)	1002/6091 (16.5)	43/1499 (2.9)	244/6091 (4.0)
D	≥60	53/332 (16.0)	337/612 (55.1)	23/332 (6.9)	85/612 (13.9)

[1]Data from SPARK index study are shown for comparison.

Abbreviation: AKI, acute kidney injury.

© 2021 Nishimoto M et al. *JAMA Network Open.*

eTable 3. Comparison of the Regression Coefficients Between NARA-AKI Cohort and the Discovery Cohort of SPARK Index in the Proportional Odds Model

	NARA-AKI cohort	SPARK discovery cohort[1]
	Coefficient (95% confidence interval)	Coefficient (95% confidence interval)
Age (vs <40)	–	–
≥40 to <60	0.272 (−0.195 to 0.740)	0.522 (0.353 to 0.691)[2]
≥60 to <80	0.178 (−0.269 to 0.625)	0.852 (0.686 to 1.019)[2]
≥80	0.064 (−0.513 to 0.642)	1.203 (0.962 to 1.443)[2]
Men (vs women)	0.341 (0.098 to 0.584)[2]	0.705 (0.616 to 0.794)[2]
Diabetes (vs none)	0.238 (−0.058 to 0.535)	0.347 (0.227 to 0.467)[2]
Expected surgical duration (continuous, hours)	0.032 (−0.032 to 0.096)	0.459 (0.433 to 0.484)[2]
Emergency surgery	0.012 (−0.450 to 0.473)	0.678 (0.441 to 0.915)[2]
RAAS blockade use (vs none)	0.123 (−0.170 to 0.422)	0.506 (0.375 to 0.638)[2]
eGFR (vs ≥60 mL/min/1.73 m^2)	–	–
≥45 to <60	0.446 (0.099 to 0.794)[2]	0.690 (0.561 to 0.818)[2]
≥30 to <45	1.143 (0.706 to 1.579)[2]	1.345 (1.141 to 1.549)[2]
≥15 to <30	1.267 (0.699 60 1.835)[2]	2.012 (1.705 to 2.319)[2]
Anemia (vs none)	0.399 (0.131 to 0.666)[2]	0.319 (0.221 to 0.418)[2]
Hypoalbuminemia (vs none)	0.740 (0.399 to 1.081)[2]	0.705 (0.590 to 0.820)[2]
Hyponatremia (vs none)	0.071 (−0.486 to 0.627)	0.298 (0.156 to 0.441)[2]
Albuminuria (vs none)	0.505 (0.173 to 0.836)[2]	0.510 (0.399 to 0.621)[2]

[1]Data from SPARK index study are shown for comparison.

[2]denotes $P < 0.05$

Abbreviations: RAAS, renin-angiotensin-aldosterone system; eGFR, estimated glomerular filtration rate.

© 2021 Nishimoto M et al. *JAMA Network Open*.

eFigure. The Receiver Operating Characteristic Curves and Calibration Plots for Postoperative Acute Kidney Injury and Critical Acute Kidney Injury From Cross-Validation in NARA-AKI Cohort Study

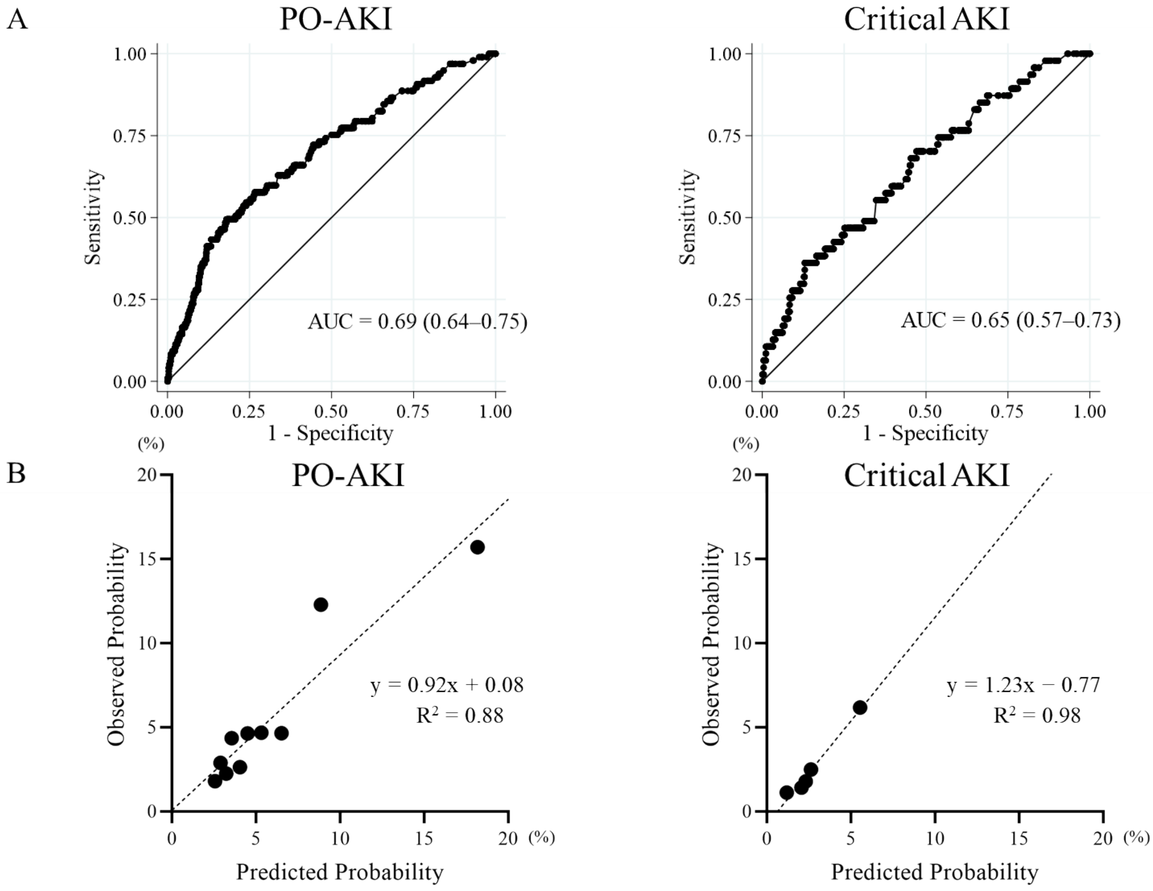

© 2021 Nishimoto M et al. *JAMA Network Open*.
